# Emerging insights into HAUSP (USP7) in physiology, cancer and other diseases

**DOI:** 10.1038/s41392-018-0012-y

**Published:** 2018-06-29

**Authors:** Seemana Bhattacharya, Dipankar Chakraborty, Malini Basu, Mrinal K Ghosh

**Affiliations:** 1 Signal Transduction in Cancer and Stem Cells Laboratory, Division of Cancer Biology and Inflammatory Disorder, Council of Scientific and Industrial Research-Indian Institute of Chemical Biology (CSIR-IICB), 4 Raja S.C. Mullick Road, Kolkata- 700032 and CN-06, Sector-V, Salt Lake, Kolkata, 700091 India; 2 Department of Microbiology, Barrackpore Rastraguru Surendranath College, 6 Riverside Road and 85 Middle Road, Barrackpore, Kolkata 700120 India

## Abstract

Herpesvirus-associated ubiquitin-specific protease (HAUSP) is a USP family deubiquitinase. HAUSP is a protein of immense biological importance as it is involved in several cellular processes, including host-virus interactions, oncogenesis and tumor suppression, DNA damage and repair processes, DNA dynamics and epigenetic modulations, regulation of gene expression and protein function, spatio-temporal distribution, and immune functions. Since its discovery in the late 1990s as a protein interacting with a herpes virus regulatory protein, extensive studies have assessed its complex roles in p53-MDM2-related networks, identified numerous additional interacting partners, and elucidated the different roles of HAUSP in the context of cancer, development, and metabolic and neurological pathologies. Recent analyses have provided new insights into its biochemical and functional dynamics. In this review, we provide a comprehensive account of our current knowledge about emerging insights into HAUSP in physiology and diseases, which shed light on fundamental biological questions and promise to provide a potential target for therapeutic intervention.

## Targeting a multi-functional protein for disease treatment

Improved understandings of a molecular-tag-removing enzyme could lead to the development of therapies for many diseases. Dr. Mrinal K Ghosh of the Council of Scientific and Industrial Research-Indian Institute of Chemical Biology (CSIR-IICB) and colleagues reviewed 20 years of research on herpesvirus-associated ubiquitin-specific protease (HAUSP), involved in a wide range of cellular processes through its role in removing the ubiquitin from molecules, thus signaling their fate. It was first discovered in/as a herpes virus infected cells, ultimately enhancing infection. It was later found to have a wide range of functions depending on the molecules it interacts with under normal physiological and disease conditions. Targeting HAUSP with drugs shows promise for suppressing prostate, lung, colon, breast, blood, and other cancers. It could also impact treatment of neurological conditions such as Huntington’s disease, and metabolic disorders, such as diabetes.

## Highlights

- A brief historical background on the discovery, the chemical and structural details of USP7/HAUSP.
- Pathological disorders arise due to HAUSP dysfunction in different physiological conditions.
- p53-dependent and p53-independent roles of HAUSP and its multiple interacting partners and substrates.
- Regulation of the multifaceted protein HAUSP and plausible pharmacological interventions in different contexts.

## Introduction

**Fig. 1:**
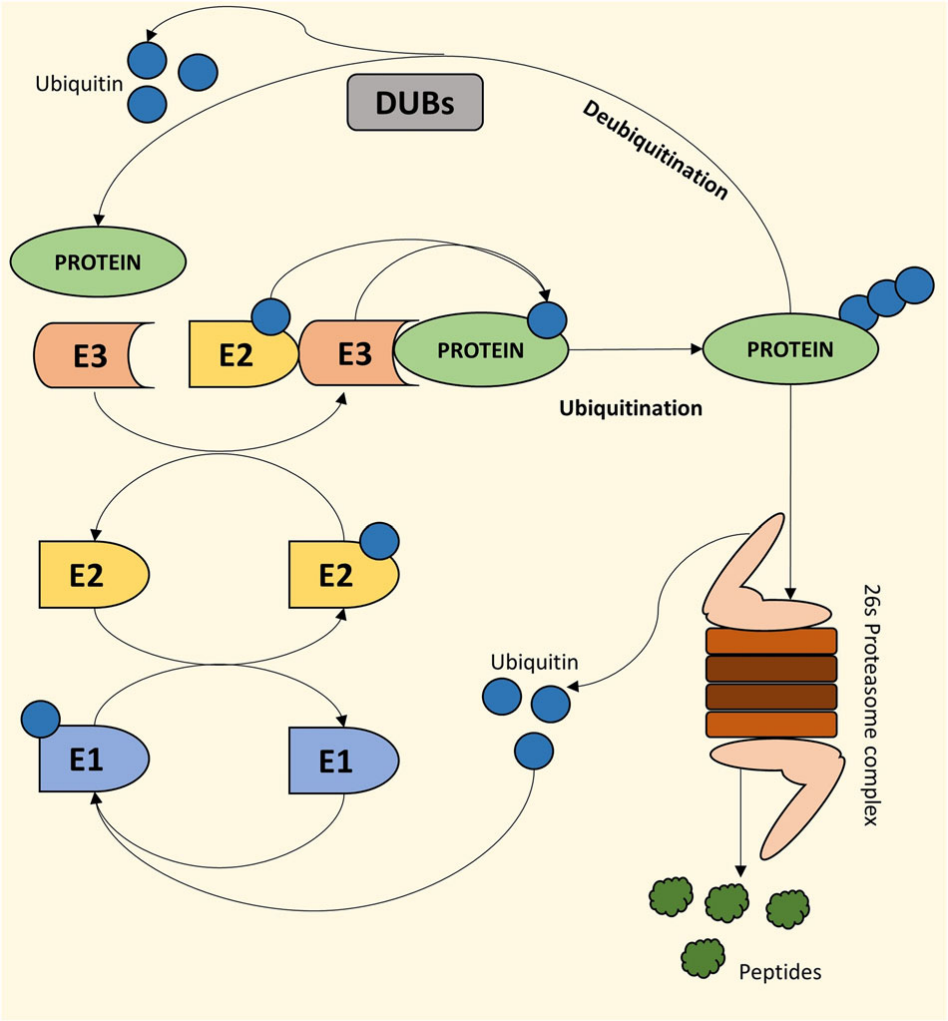
Mode of action of ubiquitin ligases and deubiquitinases for maintenance of the cellular protein pool. Pictorial representation of the 26s proteasomal degradation pathway where a series of ubiquitin ligases (E1, E2, E3) sequentially polyubiqutinate proteins for degradation. Deubiqutinases (DUBs) remove ubiquitin from proteins for their activation or escape from degradation. Altogether, these pathways maintain cellular homeostasis by regulating cellular protein pool

Aberrant changes in cellular protein expression are frequently correlated with diseases and prognosis. The cell has its own systems to maintain proteostasis, including post-translational modifications (PTMs), such as ubiquitination and deubiquitination. The seminal work of Aaron Ciechanover, Avram Hershko and Irwin Rose on the discovery of the intracellular proteolytic machinery in 1980 was followed by extensive research that has shed light on the highly regulated process of tagging proteins with ubiquitin. The ubiquitin proteasome system (UPS) utilizes a cascade of enzymes, including a few ubiquitin-activating E1s, a handful of ubiquitin-conjugating E2s and hundreds of E3 ubiquitin ligases, ensuring very specific substrate recognition (Fig. 1).^1^ Specific lysine (K) residues are involved in tagging ubiquitin (Ub) moieties, including K6, K11, K27, K29, K33, K48, K63 or Met1, each of which exhibits a specific functionality. Ub-tagged proteins may be polyubiquitinated, monoubiquitinated or even multi-monoubiquitinated, determining the fate of the protein, i.e., degradation, lysosomal targeting, signaling, or involvement in other cellular processes, such as changes in subcellular localization, membrane trafficking, histone function, transcription regulation, DNA repair, DNA replication and signal transduction.^2,3^

The processes of ubiquitination and deubiquitination are extremely dynamic, involving transient protein–protein interactions with high specificity and functioning in a highly context-dependent manner, regulating not only proteostasis but also protein function. Depending on the substrate and the context, deubiquitinases (DUBs) are implicated in controlling various cellular processes, such as chromosome segregation, DNA repair, gene expression, protein localization, kinase activation, protein degradation, cell cycle progression, and apoptosis.^2,4–6^ DUBs are involved in processing ubiquitin precursors, de-novo ubiquitin synthesis, cleavage of polyubiquitin chains, recycling of ubiquitin, and reversal of ubiquitin conjugation.^4,7^

Herpesvirus-associated ubiquitin-specific protease (HAUSP), which is also known as ubiquitin-specific peptidase or protease 7 (USP7), was discovered, cloned and characterized by Everett et al.^8^ as a 135 kDa cellular protein. The HAUSP gene is located at chromosome 16, specifically gene locus 16p13.3.^9^ HAUSP protein can bind to the herpes simplex virus type 1 (HSV-1) regulatory protein, which is known as infected cell polypeptide 0 (ICP0). HAUSP exhibits a discrete punctate pattern that is visualized as nuclear foci within the nuclear bodies (NBs) known as nuclear domain 10 (ND10), promyelocytic leukemia nuclear bodies (PML-NBs) or PODs. These structures are enriched during the initial phase of HSV-1 infection.^10^ As a consequence of its interaction with HAUSP, ICP0 migrates to the PML-NBs and causes their disruption to reactivate the viral lytic cycle. Although no specific substrate of HAUSP was identified at that time, it was hypothesized to have a regulatory role in viral and cellular gene functions.^8,11^

We currently know that HAUSP is involved in a diverse array of cellular processes. Accumulating evidence indicates that HAUSP plays critical roles in cancers, neurological disorders, metabolic disorders, immune dysfunction, etc. The first substrate identified for HAUSP-mediated deubiquitination was the tumor suppressor protein TP53 (p53) by Li et al.^12^ A tumor suppressive role was attributed to HAUSP given its ability to increase the half-life of p53, resulting in growth repression and reactivation of apoptotic pathways. Later, mouse double minute 2 homolog (MDM2) was also found to be regulated by HAUSP-mediated deubiquitination, leading to degradation of p53 and reversal of the above mentioned cellular phenotype.^13,14^ This dynamic nature of deubiquitination leading to a change in substrates was determined by the amount of genotoxic stress in cells, demonstrating that HAUSP activity is highly context-specific, exhibiting its versatility in substrate selection (discussed later). Subsequent research led to revelation of numerous additional HAUSP substrates and demonstrated that HAUSP exhibits both tumor suppressive and oncogenic properties. These findings make HAUSP an attractive target for pharmacological discoveries and in the design of specific treatment strategies.

**Fig. 2:**
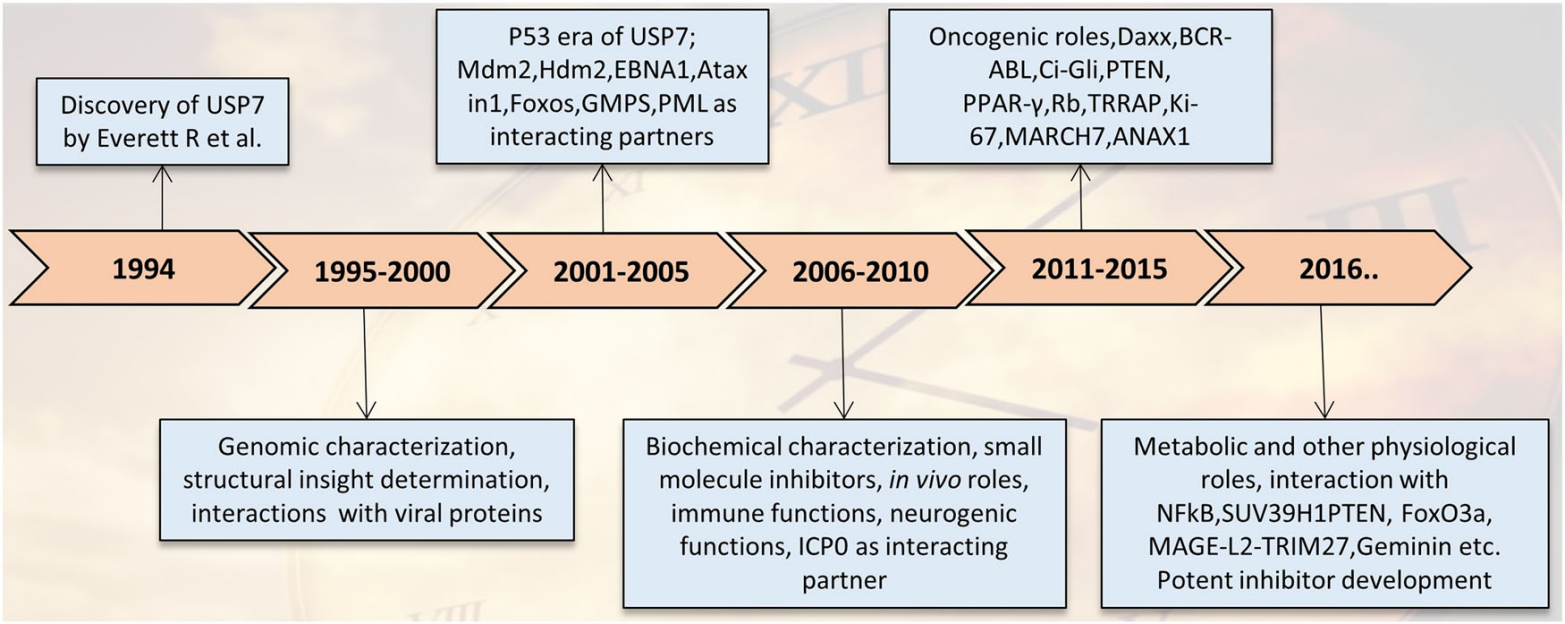
The HAUSP timeline: 20 years of research. A schematic representation of the 20 years of research on HAUSP from its discovery to the current findings and the relevant discoveries in different phases

In this review, a comprehensive account of our current knowledge to document emerging insights into HAUSP in physiology and cancer is sequentially discussed in detail. First, we provide a brief historical background on the discovery and the chemical and structural details of the molecule (Fig. 2). Second, we discuss different physiological conditions under which HAUSP functions and the pathological disorders associated with its dysfunction. Third, we elaborate on the interacting partners and substrates of HAUSP, focusing on both p53-dependent and -independent roles. Finally, we focus on the regulation of HAUSP protein and plausible pharmacological interventions utilizing this molecule in therapeutic strategies in different contexts.

## Properties and domain structure of HAUSP protein

Some of the initial research on HAUSP sought to elucidate the properties of the molecule, viz., its chemical nature, enzymatic activity, catalytic properties, and conformations in the cellular milieu. These studies were followed by analysis of the protein from an evolutionary perspective and details of the domain structures and the functional significance of each of them.

### HAUSP properties

HAUSP is evolutionarily conserved and exhibits significant (~98.6%) amino acid sequence homology with rat and mouse proteins.^15^ HAUSP is an ubiquitin hydrolyzing enzyme belonging to the ubiquitin-specific protease (USP) family.^16^ Based on its chemical nature and similar to all USPs, HAUSP is a cysteine protease that mediates the thiol-dependent hydrolysis of ester, thioester, amide, peptide and isopeptide bonds formed by the carboxy-terminal glycine residue of ubiquitin (Ub).^15^ The IUPAC nomenclature for the enzyme is EC 3.1.2.15.

There are ~100 DUBs in the human genome, functioning in a very dynamic and specific manner. Despite being a group of proteases, on the basis of their sequence and structure similarities, the DUBs can be classified into 6 families: USPs, ubiquitin carboxy-terminal hydrolases (UCHs), ovarian-tumor proteases (OTUs), Machado–Joseph disease protein domain proteases (MJDs), JAMM/MPN domain-associated metallopeptidases (JAMMs) and monocyte chemotactic protein-induced proteins (MCPIPs). All of these are cysteine proteases except JAMMs, which are metalloproteases. The largest family of these is the USP family with >50 members.^16^

### Domain structure of HAUSP

**Fig. 3:**
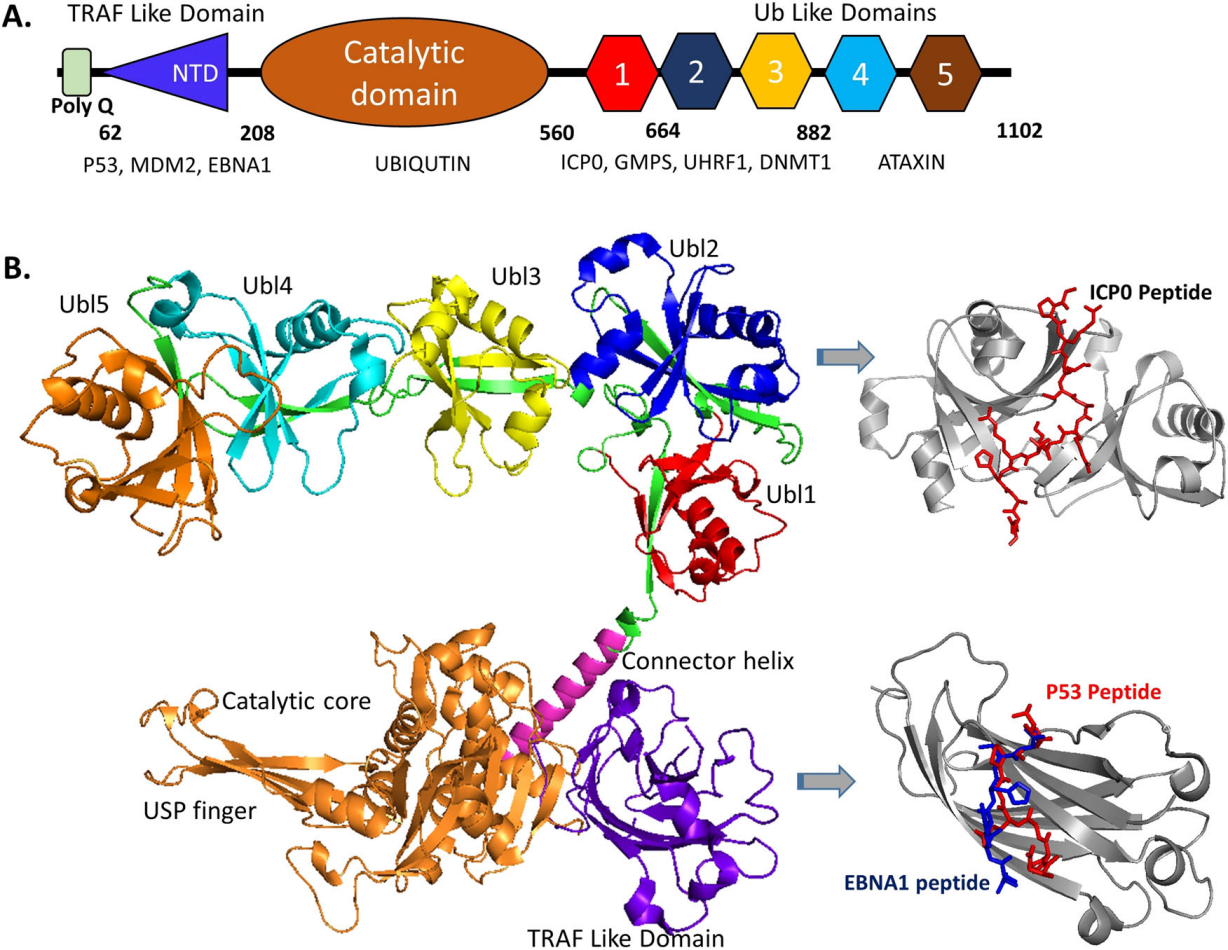
Domain structure of HAUSP. **a** Pictorial representation of HAUSP demonstrating the different domains and binding sites for major interacting partners. **b** Predictive representation of the complete HAUSP structure (by superimposing pdb structures 4yoc, 5fwi, and 2f1z) with domains representing their respective binding partners ICP0, p53, and EBNA1

The distinguishing structural property of the USPs is their highly conserved USP domain, which harbors the catalytic and ubiquitin-interacting sites. HAUSP is no exception to this. Its domains include an amino-terminal poly-glutamine (poly Q) stretch followed by the tumor necrosis factor receptor-associated factor (TRAF)-like domain, a middle catalytic (CAT) domain and a carboxy-terminal region.^15,17^ The poly Q stretch is highly conserved among human, mouse and rat. Given that the aberrant expression of CAG repeats is associated with neurodegenerative disorders, this region might have implications in neurodegeneration. However, such roles have not been associated with poly(Q) of HAUSP.^15^ The TRAF domain, which maps to amino acids 62–208, exhibits sequence homology to TRAFs and is involved mainly in protein-protein interactions utilizing the “(P/A/E)XXS” motif on the substrates. This domain also aids in nuclear localization.^18,19^ The CAT domain maps to amino acids 208–560, contains the signature amino acid sequence of the USP family of DUBs, i.e., the residues Cys, Asp(I), His and Asn/Asp(II) with the Cys residue (C223 in human) being central to the catalytic site.^20^ The carboxy-terminal domain (CTD) maps to amino acids 560–1102, contains five ubiquitin-like (UBL) folds and facilitates protein-protein interactions with ataxin-1, ICP0, ubiquitin-like PHD and RING finger domain-containing protein 1 (UHRF1), and DNA (cytosine-5-)-methyltransferase 1 (DNMT1). The CTS is a second site of interaction for p53 and MDM2 and encompasses the protein’s complete deubiquitinating activity^17,21^ (Fig. 3).

## HAUSP—physiology and disease context

Given the increase in the identified interacting partners of HAUSP, the different cellular contexts in which HAUSP functions are becoming increasingly complex. A protein found initially in the nucleus^8^ was subsequently shown to exhibit a dynamic overall cellular distribution under different pathological conditions and genetic perturbations.^22^ These context-dependent changes reflect the indispensable role of HAUSP in a physiological context, as discussed in detail later.

### HAUSP in the cellular context in vitro and in vivo

At the cellular level, HAUSP is mainly found in the nucleus and is particularly abundant in PML-NBs;^8^ however, HAUSP is also found in the cytoplasm.^23,24^ HAUSP also localizes to the mitochondria.^25^ At the tissue level, HAUSP is widely expressed in all tissue types. Under pathological conditions, for example, in cancers, HAUSP exhibits profound variation in expression in bladder,^26^ prostate,^27^ colon,^22^ lung,^28^ liver,^29,30^ ovary,^31^ brain,^32,33^ breast,^34^ etc. Recently, we also demonstrated the involvement of HAUSP in glioma progression.^23^

In the context of cancer, no effective mutations in the HAUSP gene have been identified to date, suggesting that its loss or gain of function may not be favorable for tumor growth.^35,36^ To further support this fact, in a mouse model, HAUSP knockout in vivo stabilizes p53, leading to growth arrest and embryonic lethality within 6.5–7.5 days. This effect could be partially rescued by nullifying the p53 background.^36^ This finding also signifies the importance of HAUSP during the course of mammalian development. Some studies have reported that heterozygous deletion, nonsense mutations or duplications in HAUSP lead to unbalanced neuronal homeostasis and impaired function.^37^

### HAUSP in diseases

HAUSP is mainly identified in the context of cancers and virus-associated host-pathogen interactions. However, recent studies have also demonstrated its impact in physiological contexts, such as developmental, degenerative, neurological, and metabolic disorders. Other pathologies that may be related to HAUSP dysfunction include UV radiation sensitivity, bone diseases, and cardiovascular disorders.

#### Cancer

The multi-dimensional role of HAUSP is established in various cancers, including prostate cancer, lung cancer, brain cancer, colon cancer, breast cancer, epithelial ovarian carcinoma (EOC), liver cancer, and leukemia. These data support several context-specific tumor suppressive and oncogenic outcomes. HAUSP is overexpressed in human prostate cancer. More importantly, high levels of HAUSP are directly correlated with tumor aggressiveness.^27^ In contrast, Masuya et al.^28^ found that a reduction in HAUSP gene expression may play an important role in non-small cell lung cancer (NSCLC), particularly in adenocarcinomas, through p53-dependent pathways. In addition, Ki-67 stabilization by HAUSP occurs in NSCLC.^38^ Furthermore, WD-repeat protein 79 (WDR79), which is a member of the WD-repeat protein family and functions as a scaffold protein during telomerase assembly, Cajal body formation and DNA double strand break repair, interacts with HAUSP in the nuclei of NSCLC cells, subsequently reducing the ubiquitination of Mdm2 and p53 and thereby increasing the stability and extending the half-life of the two proteins.^39^ Altered expression of coiled-coil domain-containing protein 6 (CCDC6) is regulated by PTMs, and HAUSP is responsible for fine-tuning the stability of CCDC6 to maintain ATM-dependent DNA damage response and repair in the context of lung cancer.^40^ We and others have demonstrated that HAUSP expression levels progressively increase in grade I to IV glioma tumors.^32,33^ An association between HAUSP and DNMT1 is reported in colorectal cancer that accelerates oncogenesis and metastasis.^41^ Mutated tumor suppressor gene adenomatous polyposis coli (APC) plays a major role in constitutive activation of the Wnt pathway in colorectal cancers (CRC). In certain APC-mutated CRCs, pathological levels of Wnt pathway activation are maintained by deubiquitination of β-catenin by HAUSP.^42^ HAUSP interacts and cooperates with death-domain-associated protein (Daxx) in the regulation of mitosis and taxane resistance in breast cancer.^43^ HAUSP regulates DNA replication by controlling geminin protein stability and is implicated in geminin dysregulation during breast cancer progression.^44^ HAUSP promotes deubiquitination and stabilization of the histone demethylase PHF8, leading to the upregulation of a group of genes, including cyclin A2, which are critical for cell growth and proliferation in breast cancer.^45^ MARCH7, which is ubiquitinated, acts as a potential substrate of HAUSP in EOC.^31^ In leukemias, the nuclear-cytoplasmic shuttling of PTEN plays a crucial role in disease progression. This process is fine-tuned by driver mutations, such as nucleophosmin (NPM1) and BCR-ABL.^46,47^

The large repertoire of HAUSP viral substrates exemplify the role of HAUSP in various virus-associated cancers, such as Kaposi’s sarcoma,^48^ lymphomas,^49^ and nasopharyngeal cancer.^50^ These co-occurrences of HAUSP with viral proteins in the regulation of viral lytic and latency phases underscore its importance in host-virus interactions and immunity.^51^

#### Neurological disorders

The presence of poly-glutamine repeats in HAUSP indicates its possible link to neurodegenerative disorders, such as spinocerebellar ataxias, Huntington’s disease, spinobulbar muscular atrophy, dentatorubral-pallidoluysian atrophy, and Machado–Joseph disease.^15^ In addition, its function in stabilizing repressor element 1-silencing transcription factor (REST) and maintenance of neural stem/progenitor cells connects HAUSP to neural cancers, such as medulloblastoma and neuroblastoma, and other neurological pathologies.^16,52^ Indeed, recent findings suggest that haploinsufficiency or duplications of HAUSP disrupt neuronal homeostasis, giving rise to neurological and behavioral abnormalities, such as seizures, aggressiveness, hypotonia, intellectual disability, hypogonadism with clinical features of autism spectrum disorder and a condition similar to Schaaf–Yang syndrome.^37^ HAUSP is also related to fragile X syndrome given its stabilization of PHD finger protein 8 or Jumonji C domain-containing histone demethylase 1F (PHF8).^45,53^

#### Metabolic disorders and diabetes

Peroxisome proliferator-activated receptor-gamma (PPAR-γ) is stabilized by HAUSP and plays a central role in adipogenesis, glucose homeostasis, lipid metabolism, and osteogenesis.^54^ Fine-tuning of nuclear forkhead box protein O1 (FoxO1) by HAUSP-mediated deubiquitination is also important in maintaining hepatic glucose levels. Given that these molecules are crucial regulators of functions in other metabolic tissues, such as in skeletal muscles and adipose tissues, these findings demonstrate the involvement of HAUSP in diabetes and other metabolic disorders, which makes it a suitable candidate to be targeted in the context of these diseases.^55^

#### Heart disease

In cardiac stress, MDM2 monoubiquitinates FoxO4 to increase its nuclear localization and transcriptional activity, which is reversed by HAUSP-mediated deubiquitination and nuclear export. The FoxO target atrogin-1 ubiquitinates and degrades calcineurin, preventing Akt-mediated hypertrophic signaling, and atrogin-1 also acts as a FoxO coactivator through noncanonical polyubiquitination to inhibit Akt-mediated signaling.^56^

#### Bone disease

Some reports indicate that HAUSP helps osteogenic differentiation of human adipose-derived stem cells (hASCs), but a detailed mechanism has not been established to date.^57^

#### UV sensitivity

Monoubiquitinated PCNA activates error-prone DNA polymerases, and this process is reversed by HAUSP to suppress UV-induced mutagenesis involving cell cycle independent processes, such as DNA repair.^58^ On the other hand, as part of a UV-induced ubiquitinated protein complex, UVSSA (formerly known as KIAA1530) is implicated in stabilizing the TC-NER master organizing protein ERCC6 (also known as CSB) by delivering the deubiquitinase HAUSP to TC-NER complexes. Thus, UVSSA-HAUSP-mediated stabilization of ERCC6 represents a critical regulatory mechanism of TC-NER in restoring gene expression.^59^

## Role of HAUSP in multiple cellular events

HAUSP function was first identified in the context of viral infection. Then, its p53-associated activities were extensively characterized. Several additional p53-independent functions of HAUSP were subsequently established. Several HAUSP substrates and interactors, such as viral proteins (ICP0^8^ and Epstein-Barr nuclear antigen 1 (EBNA1))^50^ and cellular proteins (PML;^60^ testis-specific protein, Y-encoded-like 5 (TSPYL5);^34^ phosphatase and tensin homolog (PTEN);^27^ and FoxOs),^61^ utilize the UPS via different strategies to perturb normal cellular functions, ultimately leading to pathological conditions. Some of these functions are indeed overlapping and interdependent; nonetheless we have attempted to broadly categorize the roles of HAUSP into different classes as discussed below (Table 1).

**Table 1:** Substrates of HAUSP

Interacting partners	Functional and pathological consequences	Disease context
p53,^12^ MDM2,^13,14^ DAXX,^43,94,95^ Rb,^33^ TSPYL5,^34^ Nucleolin,^96^ STIP,^97^ HIF-1α,^85^ Ki-67,^38^ WDR79,^39^ Geminin.^44^	Regulation of cell growth, apoptosis and tumorigenesis, hypoxia.	Cancer—breast, colon, lung, osteosarcoma, etc.
ICP0,^10^ EBNA1,^50^ vIRF1,^48^ vIRF4,^63^ LANA,^64^ UL35 and UL35a,^64^ E1B-55K,^65^ NFκB,^87^ FoxP3,^88^ TRAF6,^24^ NEMO,^24,91^ Nek2.^92^	Host-virus interactions, inflammation, immune functions, and cancer signaling	Viral infection and associated cancers—Burkitt’s lymphoma, Hodgkin’s lymphoma, HSV-1 infection, Kaposi’s sarcoma, nasopharyngeal carcinoma, etc.
PPAR-γ,^54^ FOXO1.^55^	Regulation of adipogenesis, osteogenesis, glucose and lipid metabolism	Metabolic disorders and diabetes
Ci/Gli,^90^ REST.^52^	Positive regulation of Hedgehog signaling; neurodevelopment; development	Development, cancer and neurodegeneration
Cry1 and 2,^68^ Chk1,^69^ Claspin,^70^ Chfr,^71^ MCM-BP.^69^	Regulation of genome integrity and stability	Development, cancers—colon, glioma, osteosarcoma, etc.
Tip60,^72^ CCDC6,^40^ RNF168,^74^ ANXA1,^75^ Polη,^58^ Rad18,^75^ PCNA,^58^ UVSSA and ERCC6,^59^ PHF8.^45^	Replication, DNA damage signaling and DNA repair processes	UV-sensitive syndrome and cancers, oxidative stress, DNA damage, etc.
MEL18 and BMI1,^77^ GMPS,^78,90^ DNMT1,^41,79–81^ ASXL1,^82^ AR,^83^ SUV39H1,^84^ Rae1, Bub3 and Nup98.^36^	Epigenetic regulation—chromatin dynamics and gene expression	Cancers—breast, cervical, colon, lung, prostate, MDS, etc.
PTEN,^27,46,47^ FoxO3a and 4,^61^ FoxO6,^100^ PML,^60^ TRRAP,^98^ TRIP12,^30^ MEX-3C,^99^ β-catenin,^42^ RNF220,^93^ MARCH7.^31^	Modulation of cellular signaling for tumorigenesis or tumor suppression	Cancers—epithelial ovarian, hepatocellular, leukemia, lung, osteosarcoma, prostate, etc.
Alix/Hp95,^89^ MAGE-L2-TRIM27- WASH.^37^	Endosomal protein trafficking	Multiple cancers

Table showing different substrates of HAUSP, the functional and pathological consequences

### HAUSP and viruses

**Fig. 4:**
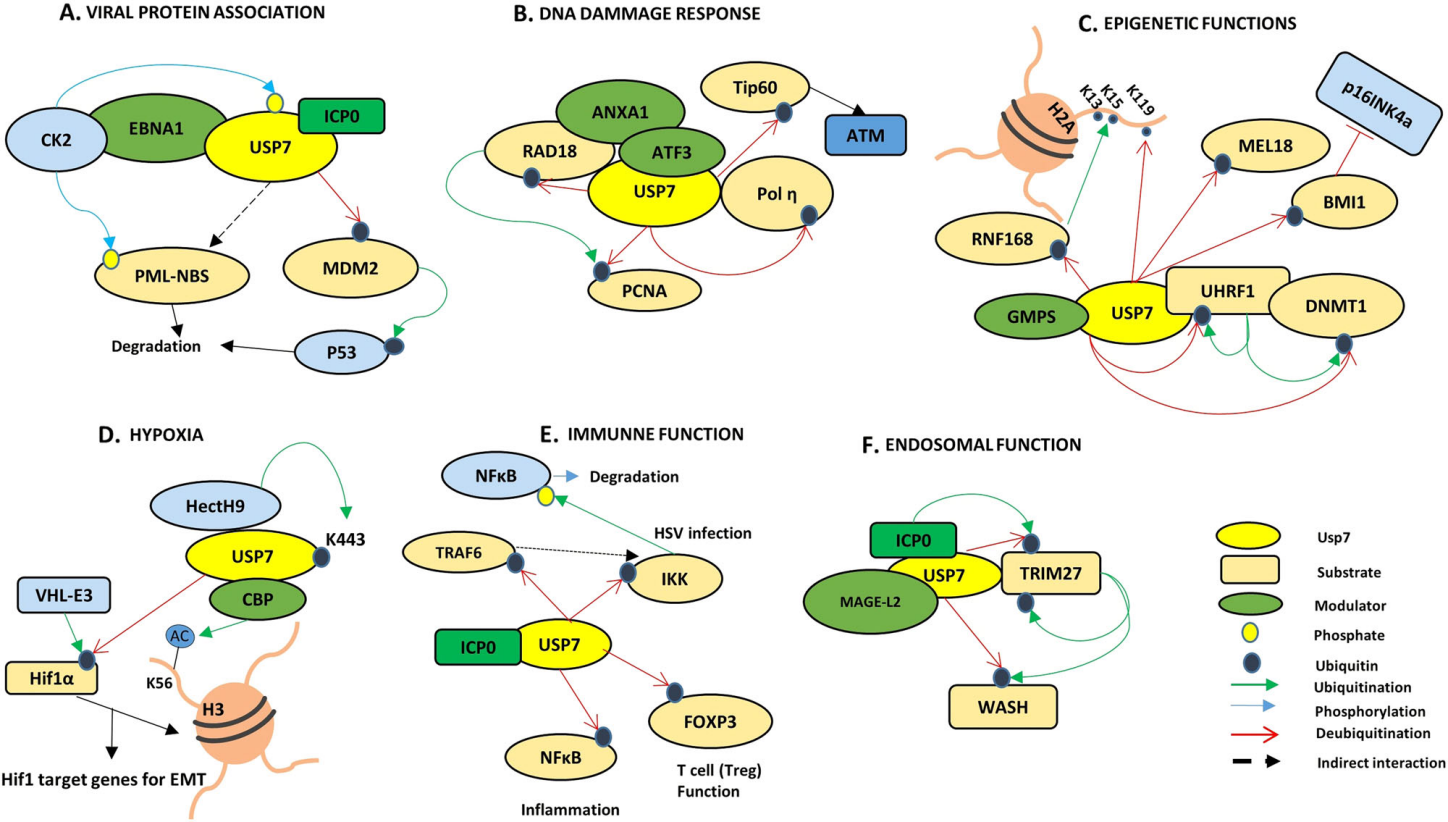
Role of USP7/HAUSP in multiple cellular events. Figure depicts involvement of USP7/HAUSP in multiple cellular events (**a** Viral-protein association; **b** DNA damage response; **c** Epigenetic functions; **d** Hypoxia; **e** Immune functions; and **f** Endosomal functions)

As discussed above (in section 1), HAUSP was identified as a factor interacting with HSV-1 early protein ICP0.^9^ In addition, EBNA1 of Epstein Barr Virus (EBV),^50^ viral interferon regulatory factor 1^48^ and 4^62^ (vIRF1 and vIRF4) and latency-associated nuclear antigen (LANA)^63^ of Kaposi’s sarcoma herpes virus, UL35, and UL35a of cytomegalovirus,^64^ and E1B-55K of adenovirus^65^ also interact with HAUSP. These findings suggest that HAUSP might be a common target of herpes viruses. The interactions of HAUSP with the different viral proteins lead to the disruption of the tumor suppressor functions of p53. This notion is exemplified by EBNA1, which, similar to MDM2, competes for the p53-binding site on HAUSP, ultimately enhancing viral infection. EBNA1 exhibits tenfold greater affinity towards HAUSP compared with p53^19^ (Fig. 4a). The sequestration of HAUSP by viral proteins perturbs its normal cellular function, thus evading cell immunity and facilitating viral infection.^19^ A genome-wide association study in Jinghai yellow chicken reveals an association of HAUSP with two avian disease viruses, Newcastle disease virus and infectious bronchitis virus, suggesting a potential role of HAUSP in generating protective antibodies against these viruses.^55,66^

### DNA dynamics

Genomic dynamics include various changes in DNA and chromatin structures, conformations, DNA damage and repair systems, large molecular complexes, and epigenetic changes that facilitate variations in gene expression and a variety of cellular responses to different cellular contexts. HAUSP plays an important role in many of these functions, as discussed below (Fig. 4b).

#### Circadian rhythm: DNA replication, cell cycle and genome integrity

Genomic integrity is maintained by a number of factors, including the circadian transcriptional repressors cryptochrome 1 and 2 (Cry1 and Cry2), which have evolved from bacterial light-activated DNA repair enzymes (phytolyases). In response to genotoxic damage, Cry1 is phosphorylated and deubiquitinated by HAUSP, whereas Cry2 undergoes Fbxl3-mediated degradation. These responses lead to an increased Cry1 to Cry2 ratio that controls the circadian rhythm and hence the downstream transcriptional response.^67^

Recently, HAUSP has been found to associate with the minichromosome maintenance complex (MCM) through the MCM-binding protein and is involved in unloading the MCM complex at the replication fork, acting as a SUMO-deubiquitinase. The lack of HAUSP decelerates progression through the S phase due to improper MCM unloading on the chromatin.^68^

HAUSP deubiquitinates checkpoint kinase Chk1,^69^ its activator Claspin,^70^ and checkpoint with forkhead and RING finger domain protein (Chfr).^71^ These proteins are important in maintaining overall genome integrity and stability.^9^

#### DNA damage

Activating transcription factor 3 (ATF3) promotes the interaction between HAUSP and Tat interactive protein 60 (Tip60).^72^ Stabilized Tip60 deacetylates DNA damage kinase Ataxia telangiectasia mutated (ATM), triggering the DNA damage response pathway during UV-irradiation stress.^73^ Another downstream effector in the ATM pathway is CCDC6, which is also subject to fine-tuning of expression by HAUSP-mediated stabilization via counteracting E3 ligase FBXW7.^40^ HAUSP modulates the stability of three E3 ligases, including RING finger protein 168 (RNF168),^74^ to regulate histone H2A and participate in Ub-dependent DNA damage signaling.

Annexin a1 (ANXA1) exhibits enhanced affinity for HAUSP upon UV-induced DNA damage; however, the response to HAUSP-mediated deubiquitination is cell-type specific. In addition to the DNA damage response, ANXA1 has roles in the immune system due to its anti-inflammatory function. ANXA1 is also involved in apoptosis and associated cell clearance. Hence, the context-specific regulation of ANXA1 deubiquitination may have diverse implications.^63^ DNA polymerase eta (Polƞ), which is a polymerase involved in translesion DNA synthesis (a member of the DNA damage tolerance pathway), is a target for HAUSP-mediated deubiquitination. The functional consequence is recruitment of Rad18 and proliferating cell nuclear antigen (PCNA) at the stalled replication forks and monoubiquitination of PCNA under UV-induced stress to accomplish DNA lesion bypass for stress tolerance.^58^ Rad18 itself is also a direct target of HAUSP-induced deubiquitination, promoting efficient DNA damage bypass.^75^

#### DNA repair

HAUSP interferes with the DNA repair system. This interference is first evidenced by the response to oxidative DNA damage and modulation of DNA accessibility to base excision repair (BER) machinery, which is an indirect effect due to HAUSP-mediated MDM2 regulation.^73^ The second line of evidence involves HAUSP’s ability to interact with UV-stimulated scaffold protein A (UVSSA), a component of the transcription-coupled nucleotide excision repair machinery (TC-NER), and stabilization of ERCC6, which resumes transcription after UV-irradiation by recruiting RNA Pol II and other factors to the promoters at the site of damage. The UVSSA and HAUSP interaction itself is also important as this leads to stability of UVSSA for efficient execution of TC-NER.^76^

#### Epigenetics

The role of HAUSP in the epigenetic modification of proteins and regulation of gene expression is evident from its ability to deubiquitinate polycomb group RING finger 2 (MEL18) and BMI1 (components of the multiprotein polycomb repressive complex, PRC1), subsequently repressing the expression of the tumor suppressor p16INK4a, various homeotic genes and hormone-dependent genes.^77^ In a similar context, HAUSP also deubiquitinates monoubiquitinated histones H2A and H2B in vitro in conjunction with GMPS (a catalytic cofactor in this case), regulating chromatin remodeling.^78^ DNMT1, which is frequently overexpressed in cancers, is rescued by HAUSP from UHRF1-mediated ubiquitination.^79^ A multiprotein complex composed of HAUSP, UHRF1, DNMT1, HDAC, Tip60, and PCNA regulates DNMT1 stability and the global methylation status of genes.^80^ Methyl-CpG binding protein 4 (MBD4) directly interacts with HAUSP to recruit it to heterochromatic foci, providing an additional mode of regulating DNMT1 (Fig. 4c).^81^

Additional sex combs-like transcriptional regulator 1 (ASXL1) is an important epigenetic regulator. ASXL1 is one of the most mutated genes in leukemias and myelodysplastic syndrome (MDS) and is associated to poor prognosis in MDS patients. ASXL1 is subject to ubiquitination, which is reversed by the deubiquitinating activity of HAUSP.^82^

Recent reports also suggest the ability of HAUSP to modulate transcription by modifying chromatin complexes. In prostate cancer, HAUSP deubiquitinates and rescues androgen receptor (AR) upon androgen stimulation and subsequently upregulates AR-responsive gene expression by direct association with AR on chromatin. This activity can be effectively targeted for therapeutic purposes to treat androgen-dependent prostate cancers.^83^

Another example is the participation of HAUSP in heterochromatinization of p53-target promoters. The suppressor of variegation 3–9 homolog 1 (SUV39H1) is a methyltransferase that marks the chromatin with H3K9me3 (repressive methylation) marks. In unstressed cells, SUV39H1 is deubiquitinated and protected from MDM2-mediated degradation by HAUSP, promoting the repression of p53-responsive gene transcription.^84^ Stabilization of histone demethylase PHF8 by HAUSP modulates both epigenetics and DNA damage repair under genotoxic stress, promoting breast cancer.^45^

Some of the other interacting partners of HAUSP are involved in chromosomal segregation, mitosis, and nuclear transport. Hence, Rae1, Bub3 and Nup98 are modulators of chromatin dynamics and gene expression.^36^

### Hypoxia

Hypoxia in the tumor core induces K63-mediated ubiquitination of HAUSP. This ubiquitination activates HAUSP to reverse the ubiquitination on HIF-1α via its E3 ligase VHL. The overall outcome is HIF-1α stabilization, promoting epithelial-to-mesenchymal transition and metastasis (Fig. 4d).^85^

### Immune functions

HAUSP has been implicated in thymocyte apoptosis via its association with caspases.^86^ NFҡB, the master regulator of inflammatory and immune signaling, is also deubiquitinated and stabilized by HAUSP, resulting in increased nuclear localization and hence transcriptional activity.^87^ FoxP3, the transcription factor associated with regulatory T cell (Treg) function, is also stabilized via HAUSP-mediated deubiquitination to maintain Treg number and function (Fig. 4e).^88^

### Endosomal functions

Proteomic analysis of HAUSP knockdown identified Alix/Hp95, which downregulates endosomal organization.^89^ Melanoma antigen family protein L2 (MAGE-L2) recruits the tripartite motif-containing protein 27 (TRIM27) to the endosome, facilitating protein recycling. WASH, a member of the Wiskott-Aldrich syndrome family, is an actin nucleation-promoting factor and is crucial for the endosomal recycling process. WASH is regulated by the MAGE-L2-TRIM27 E3 ubiquitin complex. HAUSP was identified as an interacting partner in this complex, functioning as a rheostat to balance the activation levels of WASH by either directly hydrolyzing the K63-linked Ub chains of WASH or by promoting its ubiquitination-mediated inactivation by rescuing TRIM27 from its autoubiquitination (Fig. 4f).^37^

## HAUSP in oncogenesis

**Fig. 5:**
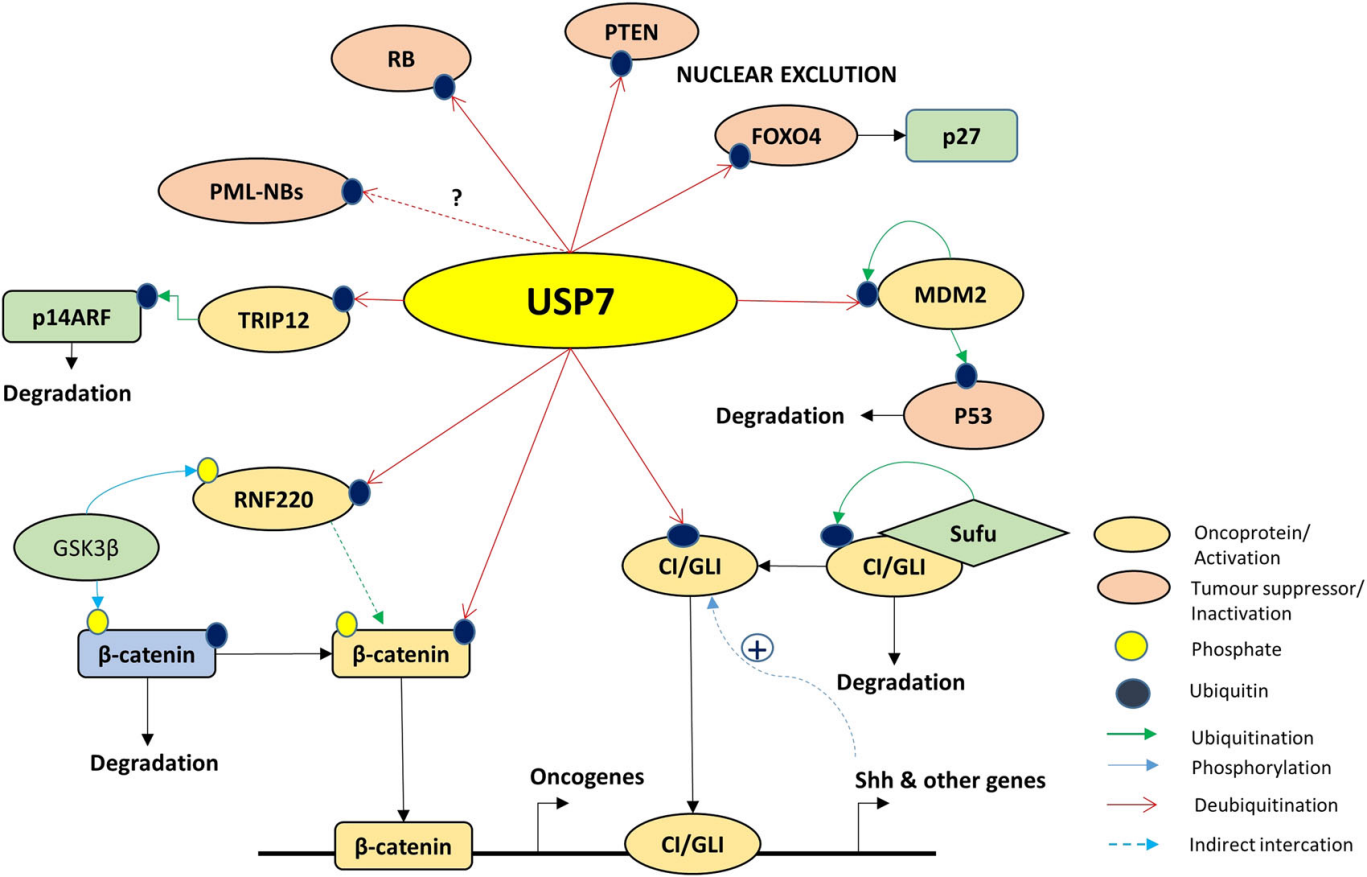
Major interacting partners/substrates of USP7/HAUSP. Figure presents various crucial interacting oncoproteins and tumor suppressors of USP7/HAUSP with various post-translational modifications (phosphorylation, ubiquitination, deubiquitination, etc.)

As mentioned in earlier sections, the role of HAUSP in the context of cancers is both tumor suppressive and oncogenic, depending on its substrates. Below, we elaborate on some of those important factors (Fig. 5).

### HAUSP and signaling dynamics

From the perspective of development, it is worthwhile to mention one of the crucial signaling pathways, namely, Hedgehog (Hh) signaling. It is a well-known pathway that contributes to embryonic development and maintains adult tissue homeostasis. Frequently, any dysregulation in this signaling pathway leads to congenital disorders and cancer. The transcription factor Ci and its mammalian homolog Gli are central players of the Hh pathway, which are tightly regulated by the processes of sequential phosphorylation and ubiquitination, targeting them to the ubiquitin proteasome system (UPS). HAUSP is the only reported deubiquitinase that rescues Ci and Gli from degradation to positively regulate Hh signaling, and this phenomenon is evolutionarily conserved in Drosophila, mammals and zebrafish.^90^ Furthermore, in another context, guanosine 5′-monophosphate synthetase (GMPS) is also a substrate for HAUSP, influencing the Hh signaling cascade. The use of inhibitors against HAUSP may provide effective outcomes by regulating the Hh pathway in Hh-related cancers.^90^

NFκB is a master regulator that is central to inflammation, immunity, cell differentiation, survival and proliferation. Thus, its activity is extremely regulated within the cell mostly by sequestration. NFκB can be directly deubiquitinated by HAUSP^87^ or can be indirectly regulated by deubiquitination of its upstream factors, such as TRAF6,^24^ NEMO (IKK-γ)^24,91^ (HSCARG interacts with NEMO to suppress its polyubiquitination by recruiting HAUSP^91^), NIMA (Never In Mitosis Gene A)-related kinase 2 (Nek2), a centrosomal serine/threonine kinase (HAUSP stabilizes Nek2 leading to activation of NFκB pathway in multiple myeloma).^92^

RNF220, a RING domain E3 ligase, regulates β-catenin stability by recruiting HAUSP to promote canonical Wnt signaling.^93^

Other pathways, such as MAPK, TGFβ-SMAD, and p53, are also regulated by HAUSP. However, these interactions are p53 dependent.

### The p53-dependent roles

HAUSP overexpression leads to p53 stabilization, whereas its partial knockdown destabilizes p53. HAUSP knockout stabilizes p53 because MDM2 is destabilized due to its autoubiquitination; thus, it cannot induce p53 degradation.^13^ Both proteins share the same interacting region, the TRAF domain on HAUSP. Thus, the dynamic role of HAUSP in switching from stabilization of MDM2/MDMX to rescuing p53 under cellular stress (genotoxic), thus promoting cell proliferation or growth arrest and apoptosis, highlights its dual or context-specific role as an oncogene or tumor suppressor.^13^ Death-domain-associated protein (DAXX) acts as an adaptor in this balancing act and is regulated by HAUSP.^94,95^ In addition to its functional relevance in balancing p53 stabilization/destabilization under different physiological conditions, HAUSP also promotes monoubiquitinated p53 in stressed cells exposed to the DNA damaging drug camptothecin^22^. Monoubiquitinated p53 can translocate to mitochondria and trigger the mitochondrial apoptotic cascade.^25^

TSPYL5 is a poor prognostic factor for breast cancer and interacts with HAUSP to impair the HAUSP-p53 interaction, suppressing p53 function and resulting in enhanced cell proliferation.^34^ This action is functionally similar to that previously reported for the viral-protein EBNA1, which competitively inhibits HAUSP-p53 complex formation.^19^

The identification of Nucleolin as a substrate of HAUSP and a member of the HAUSP-p53-MDM2 complex has added to the complexity of its dynamics, implicating cellular adaptations to combat DNA damage stress by upregulating Nucleolin in the presence of ionizing radiation.^96^ Another HAUSP-interacting nuclear factor, Sip1/Tuftelin-interacting protein (STIP), may act as a scaffold to regulate p53-MDM2 dynamics.^97^

### Regulation of oncoproteins

HAUSP is both an oncogene and a tumor suppressor in a context-dependent manner, and its substrates play major roles in its functions in either oncogenesis or tumor suppression. We previously discussed most oncoproteins regulated by HAUSP in an earlier section, so we briefly present these proteins here. HAUSP deubiquitinates REST to promote neural progenitor cell maintenance as discussed earlier.^52^ Transformation/transcription domain-associated protein (TRRAP) is another HAUSP-interacting partner, which opens up a new axis portraying the oncogenic role of HAUSP via the regulation of oncoprotein cMyc.^98^ Additional tumor-promoting roles are exemplified by the debiquitination of thyroid hormone receptor-interacting protein 12 (TRIP12), which degrades and inactivates p14ARF to promote hepatocellular carcinoma^30^ and deubiquitination of Ki-67 to promote NSCLC.^38^ The E3 ligase MARCH7 is also deubiquitinated by HAUSP, rescuing it from autoubiquitination and leading to an overall poorer prognosis in EOC.^31^ HAUSP in complex with RNF220 deubiquitinates β-catenin-enhancing canonical Wnt signaling.^93^ HAUSP interacts with the RNA-binding E3 ligase MEX-3C and antagonizes its ability to degrade mRNA. Whether this regulation occurs directly via deubiquitination of MEX-3C or indirectly through any of its substrates remains unknown.^99^

### Regulation of tumor suppressors

In this section, we will describe in detail some of the prominent substrates of HAUSP that are tumor suppressors.

#### PTEN function

HAUSP deubiquitinates monoubiquitinated nuclear PTEN, facilitating nuclear exclusion of PTEN and promoting cancer progression in prostate cancer.^27^ Changes in subcellular localization of PTEN mediated by HAUSP are crucial in certain leukemias. In NPM1-mutated acute myeloid leukemia (AML), mutated NPM1 inhibits HAUSP-mediated deubiquitination of PTEN for retention in the cytoplasm and subsequent degradation.^46^ In chronic myeloid leukemia (CML) harboring the BCR-ABL mutation, BCR-ABL promotes HAUSP-mediated deubiquitination of nuclear PTEN to drive it out of the nucleus. This activity is abolished in CML stem cells where PML expression is very high and exhibits reciprocal roles that can counteract HAUSP activity.^47^

#### FoxO proteins

FoxO4 is an important transcription factor in the Akt signaling pathway, regulating the cell cycle inhibitor p27. Cells undergoing oxidative stress also exhibit a subcellular FoxO4 shuttling strategy. Monoubiquitinated FoxO4 is deubiquitinated by HAUSP, and oxidative stress triggers the nuclear exclusion of FoxO4, negatively regulating its transcriptional activity.^61^ FoxO3a also interacts with HAUSP, suggesting that HAUSP may have a potential role in targeting the entire family of FoxO transcription factors and subsequent downstream functions.^61^ FoxO6 is downregulated in lung adenocarcinoma, and FoxO6 overexpression upregulates HAUSP and consequently facilitates p53 stabilization.^100^

#### Retinoblastoma (Rb) protein

The role of HAUSP in regulating the tumor suppressor protein of retinoblastoma (Rb) presents a unique scenario in which HAUSP maintains its level reciprocally in normal and cancer cells, and this effect crucially depends on the level of MDM2.^33^ This situation presents an opportunity to distinguish cancer and normal cells at the molecular level, providing an opportunity for targeted therapeutic strategies.

## HAUSP regulation

**Fig. 6:**
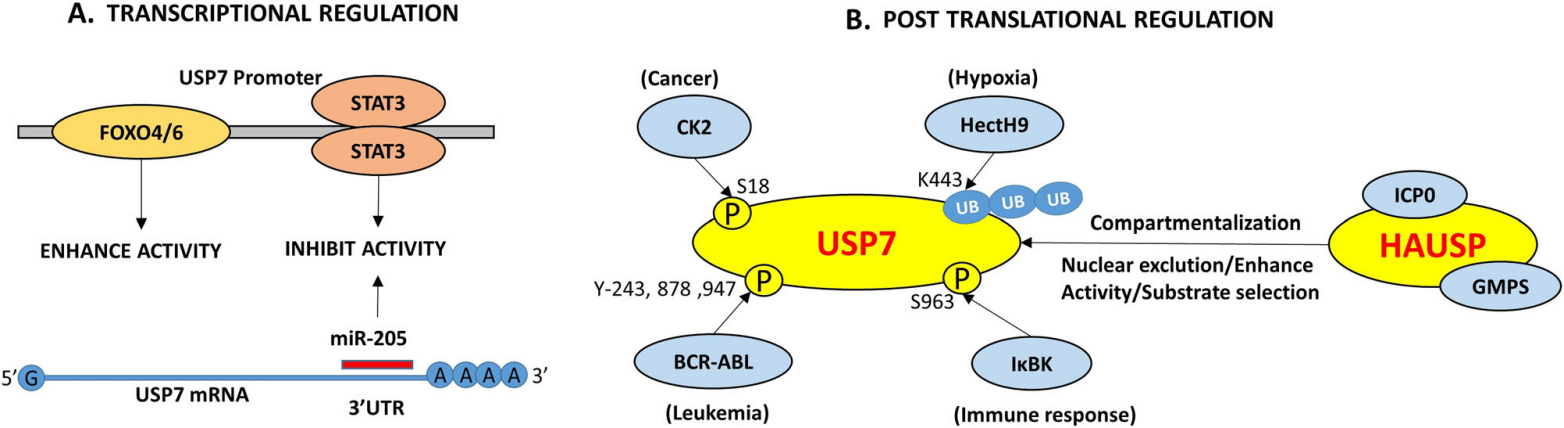
Regulation of HAUSP. A pictorial representation of the different modes of HAUSP regulation (**a** Transcriptional and **b** Post-translational)

As can be discerned through its numerous substrates, HAUSP is an important molecule involved in a number of diseases. Although it plays a multifaceted role in different physiological and pathological contexts, which are well studied, the details of its own regulation are scarce. HAUSP is subject to a number of PTMs. Similar to its substrates, HAUSP is also amenable to changes in subcellular localization. HAUSP can also be induced or inhibited by different cellular stimuli (Fig. 6).

### Transcriptional regulation

The role of IL-6-induced or constitutively activated STAT3 in negatively regulating HAUSP protein and mRNA expression in colon cancer has also been reported.^101^ HAUSP expression is enhanced by FoxO6, which inhibits proliferation of lung carcinoma.^100^ Although PHF8 is stabilized by HAUSP, it can subsequently transactivate HAUSP.^45^ Another set of endogenous cellular regulators include microRNAs (miRs), and miR-205 negatively regulates HAUSP by targeting its 3‘-UTR in hepatocellular carcinoma. Its antisense may perform the reverse action.^29^

### Post-translational modifications

HAUSP protein turnover is maintained by its interacting partner ICP0, which is also an E3 ligase.^10^ The oncogenic kinase casein kinase II (CKII) also mediates phosphorylation of HAUSP at serine 18, which results in MDM2 stabilization. This action is counterbalanced by the ATM-regulated phosphatase PPMG1, resulting in HAUSP dephosphorylation and consequently MDM2 degradation and p53 stabilization. This interaction thus establishes a network to regulate HAUSP function by balancing phosphorylation, dephosphorylation and ubiquitination.^98,102^

HAUSP’s interaction with GMPS allosterically stimulates its deubiquitinating activity.^78^ Studies with rat HAUSP reveal the presence of higher molecular weight adducts of HAUSP in the presence of ubiquitin or ubiquitin-like molecules, such as Nedd8, facilitating the ubiquitination and neddylation of HAUSP. However, no other modulator, such as Nedd8 or E3 ligases (except ICP0), has been reported in this respect, leaving this aspect of HAUSP regulation also largely unknown. Rat HAUSP forms dimers, but how this dimerization is facilitated or how it affects HAUSP functionality or even subcellular localization are not known.^103^

### Compartmentalization

The spatial distribution of proteins is crucial in determining their activities, and demonstrated how HAUSP-mediated deubiquitination of FoxOs and PTEN regulates protein function. Interestingly, studies have shown that HAUSP, which is mainly a nuclear protein, is also present at low levels in the cytoplasm and subject to shuttling between the nucleus and cytoplasm as described earlier in this review. ICP0 can export HAUSP from the nucleus to cytoplasm where HAUSP binds to and deubiquitinates K63-linked polyubiquitin chains of TRAF6 and IKK-complex, thereby inhibiting the NFκB-mediated innate immune response.^24^ On the contrary, BCR-ABL phosphorylates HAUSP (at multiple Tyrosine residues 243, 878 and 947) in the cytosol in CML and promotes its shuttling into the nuclear bodies in the nucleus, leading to PTEN nuclear exclusion by deubiquitination of monoubiquitinated PTEN and providing signals for the leukemic cells to proliferate.^47^ In addition, PML negatively regulates the function of HAUSP through DAXX to further modulate PTEN activity in a context-specific manner.^27^

## Therapeutic developments for targeting HAUSP

USPs are responsible in regulating a large number of cellular processes. Given their substrate specificity, USPs are potential targets for drug development. Similar to HAUSP, DUBs represent good targets to develop therapeutic drugs especially for diseases, such as cancer.^104^ Therefore, attempts to inhibit HAUSP activity from a therapeutic perspective have led to the development of some inhibitors that mainly boost p53 levels and induce apoptosis in cancer cells.^105^

**Fig. 7:**
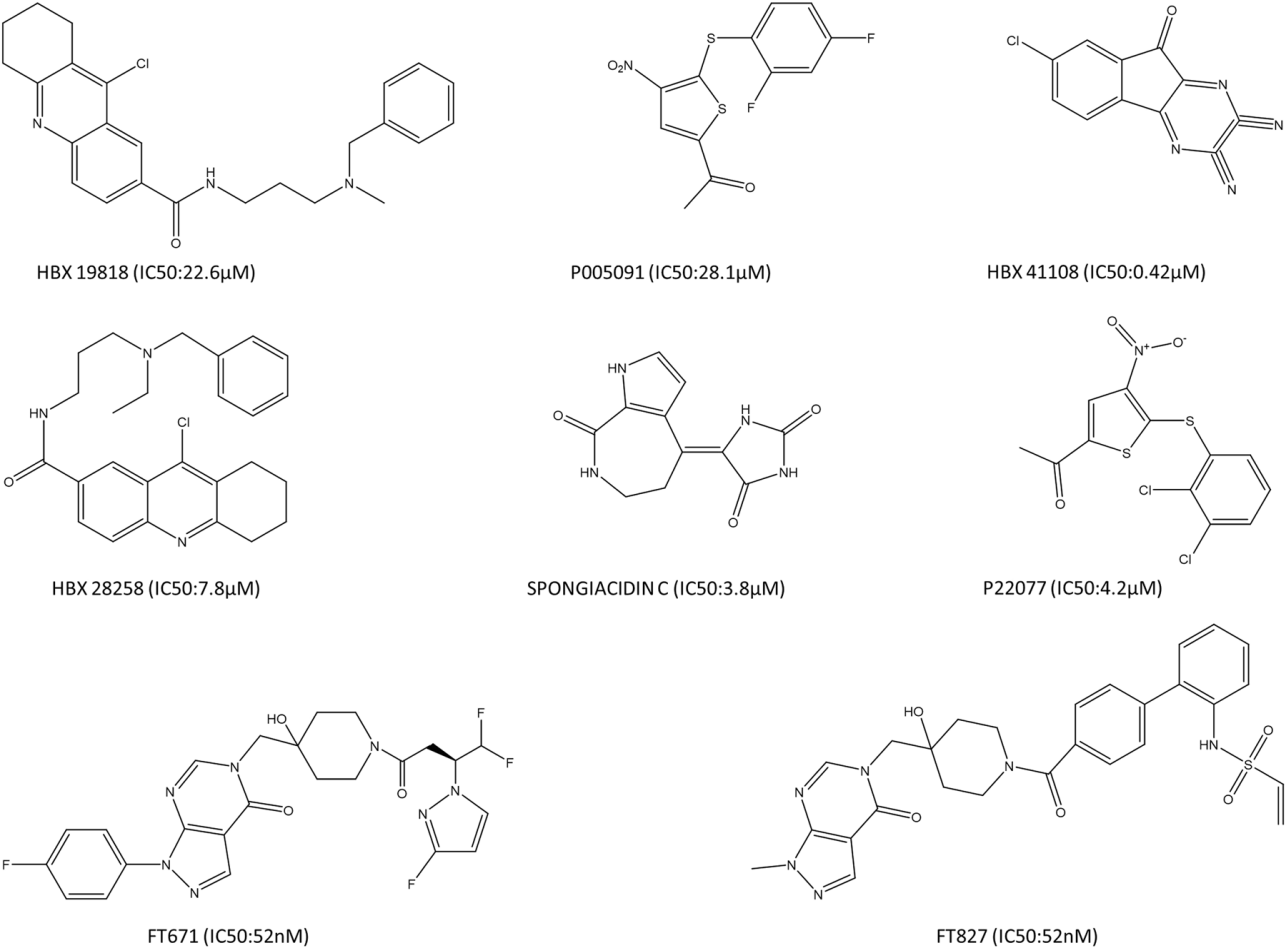
Structures and IC_50_ values of potential HAUSP inhibitors. A diagrammatic representation of the chemical structures of potential inhibitors of HAUSP with their reported IC_50_ values

Some inhibitors of HAUSP also induce endoplasmic reticulum (ER) stress due to accumulation of polyubiquitinated protein substrates in cancer cells, which leads to increased intracellular reactive oxygen species (ROS). Increased ROS are the main cause of apoptosis in these cells. All the therapeutic agents or inhibitors can be categorized as follows: small molecule inhibitors, such as HBX 41108, HBX 19818 and HBX 28258, P5091 and P22077,^104–107^ FT671 and FT827,^107^ GNE-6640 and GNE-6776;^108^ *peptides*, such as peptides against Kaposi’s-sarcoma-associated-herpes virus protein vIRF4, vif1 (aa 202-216) and vif2 (aa 220-236) (US Patent – US20140073585 A1);^62^ pre-clinical drugs, such as tyrosine kinase inhibitor targeting BCR-ABL, imatinib significantly inhibits HAUSP and beneficially upregulates p53 in Ph^+^ALL and CML.^109,110^ Clinical trials of Trisenox (arsenic trioxide), which is used for treatment of acute promyelocytic leukemia (APL), disrupts the MDM2-DAXX-HAUSP complex (reported at AACR 2016). Metformin is primarily used to regulate AMPK–mTOR signaling, inhibits HAUSP activity and enhances growth inhibition in cancer cells.^111^ The natural compound spongiacidin C (from the marine sponge *Stylissa massa*) is another therapeutic agent.^112^ Current findings related to chronic lymphocytic leukemia (CLL) suggest that the USP7 inhibitor P5091 arrests cell growth and promotes apoptosis via restoration of PTEN.^113^ Another study demonstrates that inhibition of USP7 induces genotoxic stress and DNA damage in CLL cells.^114^ Thus, these recent findings suggest that USP7 represents a promising therapeutic target in different blood-born cancers (Fig. 7).

## Concluding remarks

In conclusion, this review illustrates our current knowledge of HAUSP, an important protein with complex behavior, regulating multiple aspects of a cell in both normal and pathological states. The processes of ubiquitination and deubiquitination are extremely dynamic and context-specific. Given their specific natures, these processes are excellent targets for drug development. With the first attempt in targeting the proteasome with Bortezomib (Velcade) and subsequent discoveries of DUB-targeting compounds, this field holds a promising future. In this regard, a protein like HAUSP, which has varied implications in different cellular contexts and plays important roles in several pathologies, is also a very good target from a therapeutic point of view. Research on several inhibitors of HAUSP, including both synthetic and natural compounds, is ongoing, and recent developments are extremely promising.

More literature is now emerging on interesting patterns of E3 ligases and DUBs in complexes forming supramolecular entities in regulating cellular processes and establishing a balancing mechanism. This molecular seesaw provides the cell with ample flexibility to adjust different contexts and is tipped towards a favorable state under pathological conditions. In-depth mechanistic evaluation of such complexes can therefore be utilized especially for small molecule-mediated interventions. Therefore, further research into the regulation and role of HAUSP is expected to provide new and fundamental insights into the overall biological involvement of HAUSP in the near future.
